# A child-robot theater afterschool program can promote children’s conceptualization of social robots’ mental capacities and engagement in learning

**DOI:** 10.3389/frobt.2025.1529421

**Published:** 2025-03-14

**Authors:** Jiayuan Dong, Shuqi Yu, Koeun Choi, Myounghoon Jeon

**Affiliations:** ^1^ Department of Industrial and Systems Engineering, Virginia Tech, Blacksburg, VA, United States; ^2^ Department of Human Development and Family Science, Virginia Tech, Blacksburg, VA, United States

**Keywords:** child-robot interaction, embodied learning, social robots, human-robot interaction (HRI), STEAM education

## Abstract

Research on integrating emerging technologies, such as robots, into K-12 education has been growing because of their benefits in creating engaging learning environments and preparing children for appropriate human-robot interactions in the future. However, most studies have focused on the impact of robots in formal educational settings, leaving their effectiveness in informal settings, such as afterschool programs, unclear. The present study developed a 9-week afterschool program in an elementary school to promote STEAM (STEM + Art) education for elementary school students. The program incorporated four modules (Acting, Dancing, Music & Sounds, and Drawing), each with specific learning objectives and concluding with a theater play at the end. This program facilitated hands-on activities with social robots to create engaging learning experiences for children. A total of 38 students, aged 6–10 years, participated in the afterschool program. Among these students, 21 took part in research activities, which included answering questions about their perceptions of robots compared to other entities (i.e., babies and beetles), learning interest and curiosity, and their opinions about robots. In addition, four teachers and staff participated in interviews, sharing their reflections on children’s learning experiences with robots and their perceptions of the program. Our results showed that 1) children perceived robots as having limited affective and social capabilities but gained a more realistic understanding of their physiological senses and agentic capabilities; 2) children were enthusiastic about interacting with robots and learning about robot-related technologies, and 3) teachers recognized the importance of embodied learning and the benefits of using robots in the afterschool program; however, they also expressed concerns that robots could be potential distractions and negatively impact students’ interpersonal relationships with peers in educational settings. These findings suggest how robots can shape children’s perceptions of robots and their learning experiences in informal education, providing design guidelines for future educational programs that incorporate social robots for young learners.

## Introduction

Emerging technologies are widely integrated into K-12 education because of their positive impact on facilitating computational thinking (Angeli and Valanides, 2020; Bakala et al., 2021; Chalmers, 2018), problem-solving (Danby et al., 2018; Lee-Cultura, Sharma, and Giannakos, 2022; Lorusso et al., 2018), and engaging learning experiences (Dong et al., 2023; Jones, 2020; Lee et al., 2022). Furthermore, equipping children with the skills to navigate a rapidly evolving technological landscape is essential. Robots, an example of emerging technologies, have been increasingly integrated into our daily lives and have played an important role in various aspects of human activities. To prepare younger generations for a world where humans and robots coexist, it is crucial to guide young learners into learning how to use and interact with robots appropriately in their physical presence. Social robots offer significant educational benefits by enhancing children’s learning experiences and supporting their knowledge and skills through hands-on interactions and social cues (Johal et al., 2016; Kanda et al., 2012; Kory et al., 2017). In a review by Toh et al. (2016), the use of social robots in formal curricula for young children was found to improve their learning performance in STEM-related subjects. However, the effectiveness of using robots in diverse educational settings, such as informal afterschool programs, remains underexplored. Previous studies have even raised concerns about integrating robots into educational settings because of issues related to privacy (Sharkey, 2016) and disruption in teaching (Reich-Stiebert and Eyssel, 2016). To address these concerns, it is crucial to develop a well-designed educational program with an appropriate context. In the present study, we created a 9-week child-robot theater afterschool program to engage elementary school children in hands-on activities that promote exploration and learning in science, technology, engineering, arts, and mathematics (STEAM), providing an innovative approach to learning. We aimed to examine this program’s effectiveness by comparing children’s attitudes toward robots and learning behaviors before and after the program through structured and open-ended interviews.

## Related work

### Embodied/tangible education

Embodied cognition is a growing research area that emphasizes how cognitive processes are influenced by an individual’s physical interactions with the environment (Shapiro and Stolz, 2019). Cognition is not solely an activity of the mind but is instead distributed across the entire interactive experience, involving the mind, body, and environment (Wilson, 2002). Developing cognitive skills and the intellectual ability to acquire and apply new concepts is crucial in a child’s elementary school years. Tangible learning has been shown to increase accessibility for young learners and foster collaborative creation (Lee, 2016). Manipulating and interacting with tangible media, such as tablets, technological devices, and robots, fosters an environment where children can receive continuous feedback, supporting their problem-solving and decision-making skills (González-González et al., 2019). Studies on tangible technologies have identified the benefits of robotic systems, specifically in STEAM education, where they introduce a playful dimension to learning endeavors, improve student motivation and retention, and help students develop communication and problem-solving skills (Chaldi and Mantzanidou, 2021; Pandian, 2018). While tangible learning has shown promising benefits, its long-term effects on students’ cognitive development require further research. Therefore, we designed a longitudinal study to investigate the long-term effects of tangible technology, focusing on the use of social robots in STEAM education environments.

### Robots and AI systems in educational settings

Robotic systems used in education include robot platforms, robotic kits, and programmable robots (Karim et al., 2015). Robotics can provide both a platform and a tool for children to engage in robot interaction while learning how to work alongside automation and technology. In addition, the use of robots in classrooms provides insights into human-robot interaction research, particularly in understanding how young learners engage with robotic technology. Studies have shown that educational robots, such as robot arms and LEGO robots, help both teachers and students develop problem-solving skills and improve learners’ attitudes and interests in STEM-related fields (Mwangi et al., 2022). A study by Khanlar (2013) showed that simple robot kits facilitated students’ learning in STEM subjects due to their hands-on nature, their positive effects on students’ self-confidence, and their “play and learn” approach. These kits enabled students to develop their conceptual understanding and enhance their problem-solving skills. While robot kits support cognitive development, social robots have the potential to play a distinct role in fostering children’s emotional and social development while creating engaging learning activities. Previous studies suggest that using social robots in informal learning environments sustains children’s learning engagement and enhances their interest in STEAM (Dong et al., 2023; Lee et al., 2022; Barnes et al., 2020). Further, Belpaeme et al. (2018) meta-analysis found that the physical presence of social robots enhances students’ cognitive and affective outcomes. Therefore, it is important to select the appropriate type of robotic system based on specific educational objectives. Despite the positive effects and potential benefits of robotics in education, it is important to recognize that not all students learn effectively through this technology. This underscores the importance of understanding individual differences in children’s prior knowledge, skills, and beliefs before introducing them to the complexities of robotics (Barak and Assal, 2018). Toh et al. (2016) found that although parents acknowledged the benefits of educational robots for their children; they were less confident in teaching their children how to use them (Toh et al., 2016). Despite the many benefits of integrating robots into educational settings, previous research has also revealed concerns and cautious attitudes among parents and teachers. In the online study by Perella-Holfeld et al. (2024), parents and educators expressed their worries that robots might contribute to laziness in children and disrupt their peer relationships. Additionally, several studies have raised concerns about children’s physical and psychological safety in human-robot interactions, including psychological trauma and mental health (Sharkey, 2016; Vasic and Billard, 2013; Perella-Holfeld et al., 2024). Smakman et al. (2021) also addressed the moral considerations of social robots in education, highlighting privacy concerns and the methodological challenges of integrating these technologies into learning environments. Given these mixed perceptions, further research is needed to better understand how students and teachers perceive social robots in diverse real-world educational settings. To address this gap in investigating people’s attitudes toward using robots for education, we aimed to assess students’ and teachers’ perceptions and opinions on the child-robot theater afterschool program.

### Extraordinary entity: children’s perception of robots

As robots become increasingly prevalent in our everyday lives, documenting the evolving relationship between humans and robots is critical. This documentation will provide insights into the nature of human social reasoning and cognition (Weisman, 2022) and offer valuable guidance for future human-computer interaction. Prior research has addressed this topic by examining children’s perceptions of various robots, including humanoid social robots (e.g., NAO), household AI technologies (e.g., Amazon Alexa and Siri), and robots with mechanical faces, in terms of their perceived mental capacities (Flanagan et al., 2023; Gray and Wegner, 2012). For instance, Flanagan et al. (2023) revealed that children aged 4 and 11 years held different beliefs about different technologies (i.e., Roomba, Alexa, and NAO) regarding their levels of agency. It has been shown that both children and adults attribute greater physical and emotional experiences to humanoid robots than to robots with mechanical faces (Gray and Wegner, 2012).

Previous studies have found that children’s beliefs about the mental capacities of emerging technologies developed across ages (Reinecke et al., 2025; Sommer et al., 2019; Weisman, 2019). Weisman et al. (2017) compared participants’ conceptualizations of robots and beetles, two edge cases in social reasoning that challenge traditional frameworks for attributing mental capacities. Robots, though non-living entities are designed to mimic human-like behaviors, blurring the line between mechanical objects and agents capable of mental reasoning. In contrast, beetles are living organisms with limited mental abilities resembling those of humans, representing the biological extreme. In addition, babies, as humans in the early stages of development, exemplify emerging cognitive, emotional, and social capacities (Weisman, 2019; Weisman et al., 2017). Thus, beetles, as well as babies, are more likely to be perceived as being capable of biological experience, such as feeling pain, but having less agency, such as planning, compared to robots (Weisman, Dweck, and Markman, 2017; Gray et al., 2007). However, it remains unclear what environmental factors, such as learning experiences with social robots, might influence children’s conceptualization of robots’ mental capacities.

The current study examined how children’s interactions with social robots influence children’s perceptions of robots, particularly regarding mental capacities, in comparison to babies and beetles—three distinct categories of life (artifacts, humans, and animals) with varying attributions of biological and mental capabilities. The contrast between robots and beetles highlights differences in children’s perceptions of animacy and mental life, providing insight into how artificial and biological traits shape judgments (Weisman et al., 2017; Weisman, 2019). Comparing robots to babies reveals distinctions in how children perceive artificial and human mental abilities, especially advanced cognitive and social-emotional characteristics. These comparisons among the specific entities contribute to a deeper understanding of how children conceptualize robots’ mental capacities. Therefore, we sought to examine how elementary school-aged children conceptualize the mental capacities of robots, including affective, perceptual, physiological, cognitive, agentic, and social capacities. More importantly, we aimed to address the gap in the literature regarding how children’s perceptions may or may not be influenced by their educational experiences, specifically our child-robot theater afterschool program, using a longitudinal research design.

### Research questions

The present study aimed to design and evaluate an afterschool program that integrates social robots as educational tools that promote interactive, hands-on activities in STEAM education, fostering embodied learning. We designed a 9-week program comprising four modules, each designed with specific objectives incorporating STEAM-related topics and social robots for elementary school students. Based on the literature and research objectives, we developed the following research questions:

• RQ1: How will children perceive robots’ mental capacities compared to other entities (i.e., babies and beetles) before and after the program in affective experience, perceptual abilities, physiological sensation, cognitive abilities, agentic capabilities, social abilities, and awareness of things?
• RQ2: How will children’s attitudes toward social robots change before and after the program?
• RQ3: How will children’s interest, self-efficacy, and curiosity in robots and STEAM change before and after the program?
• RQ4: How will the afterschool program influence teacher’s attitudes toward using robots in informal learning settings?

To evaluate the program, children completed interviews with structured and open-ended questions about their perceptions of robots, learning experiences, and attitudes toward robots before and after the program. With this mixed-methods approach, we aimed to analyze both quantitative and qualitative responses to gain a comprehensive understanding of students’ perspectives. In addition, teachers completed interviews after the program to share their perceptions of social robots and the program. A key theoretical contribution of the present study lies in its use of measures developed by Weisman (2019). Which assesses children’s perceptions of mental capacities across multiple domains, including affective experience, perceptual abilities, physiological sensation, cognitive abilities, agentic capabilities, social abilities, and awareness of things. These measures allowed for the comparison of how children’s perceptions of robots’ mental capabilities changed before and after the program, particularly in comparison to other entities (i.e., babies and beetles). By applying this approach, the present study aimed to examine the role of social robots used in informal education in children’s conceptualization of robots’ mental capacities, which has not yet been fully explored in K-12 education and other developmental research. Based on children’s structured interview responses, the present afterschool program influenced children’s conceptual representations of robots’ mental capabilities (Weisman, 2019). In addition, qualitative interview responses from both children and teachers suggested that embodied learning, such as using social robots in informal educational settings, students’ learning experiences and outcomes. Together, the findings of the present study expand knowledge on the use of social robots in informal education and provide practical design recommendations to inform and guide researchers in developing educational programs that integrate social robots.

## Materials and methods

### Participants

The afterschool program was conducted in a Title 1 elementary school, selected based on the high proportion of low-income students. This economic criterion was determined by the Virginia Tech Center for Educational Networks and Impacts (CENI), the superintendent, and the Virginia Boys and Girls program. Children participated in the program from September to December 2022, involving a total of 38 children between the ages of 6 and 10 years (*M* = 7.6 years, *SD* = 1.39). In each module, 10–18 children participated (*M* = 14.5, *SD* = 2.88). Child assent and parental consent forms were collected before the program with the assistance of the program director at the school. Students’ attendance was not mandatory, meaning their participation may only have occurred in parts of the robot theater program sessions. Four teachers and staff participated in the program to assist the researchers with attendance roll calls and classroom discipline. Each week, the researchers set up the robots in the library space, and then afterschool teachers escorted the children to the library. Throughout the sessions, at least two to three afterschool teachers were present to facilitate children’s engagement in activities.

### Robots

Five robots were involved in the afterschool program: Pepper, NAO, Milo, Aibo, and Quincy. Social robots have been shown to be beneficial in facilitating a creative learning environment (Ali et al., 2019), we incorporated them into the present afterschool program. Social robots’ physical presence allows for emotional expressions in the program, including the final theater production, aligning with the objectives of the program involving both STEM and arts (Ali et al., 2019). Their detailed roles and responsibilities are described as follows:

### Pepper

**FIGURE 1:**
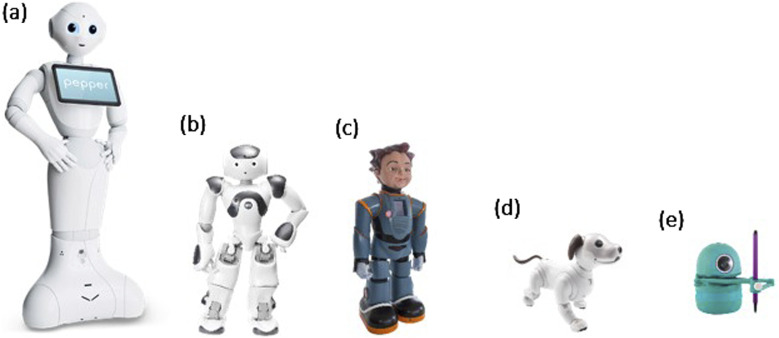
Pictures of research robots used in the present afterschool program. **(A)** Pepper **(B)** NAO **(C)** Milo **(D)** Aibo **(E)** Quiency.

(Figure 1A; Height 48 in, Length: 17 in, Width: 19 in) is a humanoid robot from Softbank Robotics. Its primary role in the afterschool program was to showcase its capabilities in providing speech and balanced movements. Children were invited to program Pepper’s animation and speech through the Choregraphe software. Pepper also provided pre-programmed games and demos to facilitate an interactive learning experience for the children.

### NAO

(Figure 1B; Height 22.6 in, Length: 12.2 in, Width: 10.8 in) is another humanoid robot from Softbank that has a smaller size compared to Pepper. With our control, NAO was programmed to act as a robot instructor every week to present half of the lectures to the students for a more engaging learning environment. During the free play session, children were also able to learn about programming NAO’s movements and speech. Nao was involved in every module of the afterschool program.

### Milo

(Figure 1C; Height 24 in) is the third humanoid robot used in the present afterschool program, developed by RoboKind. Milo’s unique features include its ability to display human facial expressions. Milo was introduced to the students to teach them about emotional expressions in theater plays, allowing children to expand their knowledge in learning robots with different appearances and capabilities. Milo’s speech, movement, and facial expressions were programmed through PuTTY, an open-source terminal emulator.

### Aibo

(Figure 1D; Height 11.5 in, Length: 12 in, Width: 7.1 in) is a robot dog developed by Sony. Aibo can recognize verbal commands from students and perform corresponding tricks. It also displayed corresponding reactions to students when they touched or “pet” Aibo’s sensors. Aibo was primarily involved in the free play sessions to teach children how to interact with robots gently and appropriately.

### Quincy

(Figure 1E; Height 6 in, Length: 4.1 in, Width: 4.1in), the robot artist, was the students’ art instructor. Students were instructed to show Quincy the QR codes for the designated drawings. There were seven Quincy robots to ensure that most students could experience hands-on drawing activities with the robots.

The research team also provided commercial robot toys for students to interact with while waiting for their turn to engage with the research robots above during free play sessions.

### Procedures/overview

The 9-week afterschool program was hosted every Wednesday from 4 PM to 5 PM at Eastern Montgomery Elementary School. Each week of the afterschool program had a specific learning goal for the students (Table 1). A pretest-posttest within-subjects design was applied to the present afterschool program. Children’s and teachers’ responses to interview questions were compared before and after conducting the afterschool program. The members of the research team who conducted the interviews with children were trained before the program following CENI’s guidance. If children struggled with answering an interview question, we rephrased the question and provided examples to aid in understanding. The scales we used in the children’s structured interview questions were a simple “no, kinda, yes” scale and a 5-point Likert scale with circles of different sizes. Further details of the interview questions are explained in the Results section. Children without assent and consent forms were still welcome to attend the program but did not participate in the research activities, such as interviews.

**TABLE 1:** Module objectives.

Weeks	Module	Goals
0 (Introduction)		• Introduce existing robot types and the robots involved in the program.
1-2	Drawing	• Discuss drawing and design involving robots. • Create drawings and costumes for the robot theater play.
3-4	Dancing	• Learn the characteristics of robots in terms of weight, balance, physicality, and finer points of robot motion. • Identify different ways to create robot motions for dancing (autonomous vs non-autonomous).
5-6	Music and Sounds	• Discuss the fundamentals of generating music and sound based on emotion and gestures using robots and AI. • Create their own sounds based on mappings with robots.
7-8	Acting	• Compare and contrast the characteristics of robots in terms of anthropomorphism. • Learn how to control robot motions and speech for acting (remote, programming, verbal commands, & autonomous decision-making).
9 (Final Performance)		• Perform a theater play using music pieces and drawings created throughout the program with social robots.

### The development of our lesson plans

**FIGURE 2:**
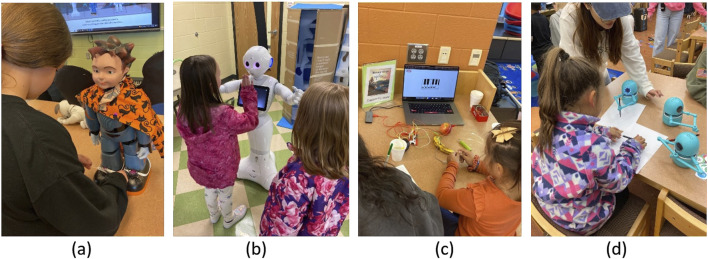
Example pictures from each module: **(A)** Acting, **(B)** Dancing, **(C)** Music and Sounds, and **(D)** Drawing.

**FIGURE 3:**
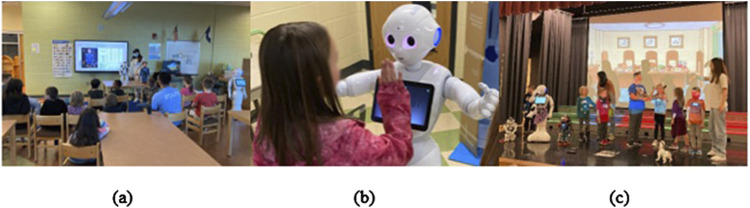
Example session activities included **(A)** educational activities, **(B)** free play, and **(C)** final theater performance.

The program included four modules, in addition to the Introduction and Performance weeks, all of which we developed prior to the start of the afterschool program. The module structures were inspired and designed by incorporating theater play elements, which were Acting, Dancing, Music & Sounds, and Drawing (Figure 2). Each session lasted an hour and consisted of a lecture (30 min) and hands-on free play (30 min). The lecture portion included two presentations, one presented by the researchers introducing the context of the STEAM topics and another by NAO, teaching children about robots’ capabilities and limitations. The room was equipped with a projector, tables, and chairs. One of the tables was designated for demonstrating robots, except for Pepper, which was placed on the floor due to its size (Figure 3).

**FIGURE 4:**
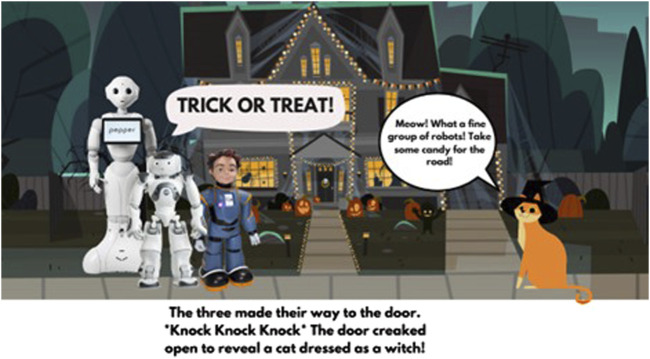
An example scene from the storybook titled “Let’s Go Trick or Treating!”.

For the purpose of this program, a storybook titled “Let’s Go Trick or Treating!” was created using Canva, a graphical software tool (Figure 4). The storybook was designed to incorporate several robots introduced in the program, namely, Pepper, NAO, and Milo. This storybook is open to public on the web for other researchers and educators: https://osf.io/7k5t3. The narrative followed a robot named Milo who went on a Halloween adventure with his robot friends, Nao and Pepper. Throughout their journey, Milo and his friends experienced various emotional states, such as fear, worry, anger, disappointment, relief, and joy. Each page of the digital storybook was projected on a screen throughout the program. Children were actively engaged with the story by constructing musical plays by creating and practicing the scripts. In addition, children shared their experiences of trick-or-treating by relating the content to their everyday lives. The story included various entities besides robots, including animals, humans, and fantasy characters, encouraging children to consider similarities and differences between social robots and other entities.

To familiarize students with the final theater play, we read the story to them each week. The story included our robots as the main characters, and they were designed with different speech lines, emotional expressions, and gestures. The story was intended to serve as a starting point for the students to expand on as they gained STEAM knowledge and skills from the afterschool program. In the final week of the program, students were invited to perform the final version of the story they developed on stage with the robots. Parents, teachers, and peers were invited to attend the play as audience members and celebrate the students’ completion of the program.

## Results

### Child pre- and post-program interviews

Children’s pre- and post-interviews with structured and open-ended questions included three parts: children’s conceptualization of entities (robots, babies, and beetles), opinions about robots, and students’ interest, curiosity, and confidence in STEAM education (Table 2). Because of the nature of the afterschool program, the sample size fluctuated between the pre- and post-interviews (*N*_*pre*_ = 16, *N*_*post*_ = 11).

**TABLE 2:** Child interview questions.

Q1	Why did you join this child-robot theater afterschool program?		
Q2	Do you think [a robot/a baby/a beetle] can...? (No, Kinda, Yes) *If this is about a robot, ask children to draw.	1. get angry	No | Kinda | Yes
		2. get hurt feelings	No | Kinda | Yes
		3. smell things	No | Kinda | Yes
		4. remember things	No | Kinda | Yes
		5. feel sick, like when you feel like you might throw up	No | Kinda | Yes
		6. feel tired	No | Kinda | Yes
		7. feel scared	No | Kinda | Yes
		8. feel embarrassed	No | Kinda | Yes
		9. sense temperatures	No | Kinda | Yes
		10. feel guilty	No | Kinda | Yes
		11. feel pain	No | Kinda | Yes
		12. feel happy	No | Kinda | Yes
		13. sense whether something is close by or far away	No | Kinda | Yes
		14. feel love	No | Kinda | Yes
		15. be aware of things	No | Kinda | Yes
		16. feel sad	No | Kinda | Yes
		17. get hungry	No | Kinda | Yes
		18. figure out how to do things	No | Kinda | Yes
		19. feel proud	No | Kinda | Yes
		20. make choices	No | Kinda | Yes
Q3	Do you think [a robot/a baby/a beetle] can...? (No, Kinda, Yes) *If this is about a robot, ask children to draw.	1. feel love	No | Kinda | Yes
		2. feel sad	No | Kinda | Yes
		3. sense whether something is close by or far away	No | Kinda | Yes
		4. smell things	No | Kinda | Yes
		5. get hurt feelings	No | Kinda | Yes
		6. figure out how to do things	No | Kinda | Yes
		7. feel sick, like when you feel like you might throw up	No | Kinda | Yes
		8. feel happy	No | Kinda | Yes
		9. get hungry	No | Kinda | Yes
		10. feel tired	No | Kinda | Yes
		11. remember things	No | Kinda | Yes
		12. get angry	No | Kinda | Yes
		13. sense temperatures	No | Kinda | Yes
		14. be aware of things	No | Kinda | Yes
		15. feel pain	No | Kinda | Yes
		16. feel embarrassed	No | Kinda | Yes
		17. make choices	No | Kinda | Yes
		18. feel scared	No | Kinda | Yes
		19. feel proud	No | Kinda | Yes
		20. feel guilty	No | Kinda | Yes
Q4	Do you think [a robot/a baby/a beetle] can...? (No, Kinda, Yes) *If this is about a robot, ask children to draw.	1. make choices	No | Kinda | Yes
		2. feel proud	No | Kinda | Yes
		3. remember things	No | Kinda | Yes
		4. feel love	No | Kinda | Yes
		5. feel pain	No | Kinda | Yes
		6. get angry	No | Kinda | Yes
		7. sense whether something is close by or far away	No | Kinda | Yes
		8. get hurt feelings	No | Kinda | Yes
		9. get hungry	No | Kinda | Yes
		10. feel sad	No | Kinda | Yes
		11. feel guilty	No | Kinda | Yes
		12. feel sick, like when you feel like you might throw up	No | Kinda | Yes
		13. be aware of things	No | Kinda | Yes
		14. feel embarrassed	No | Kinda | Yes
		15. feel tired	No | Kinda | Yes
		16. feel happy	No | Kinda | Yes
		17. smell things	No | Kinda | Yes
		18. figure out how to do things	No | Kinda | Yes
		19. feel scared	No | Kinda | Yes
		20. sense temperatures	No | Kinda | Yes
Q5	Are you excited to learn new things?	1 | 2 | 3 | 4 | 5	
Q6	Do you like to figure things out?	1 | 2 | 3 | 4 | 5	
Q7	Are you interested in things that are surprising or unusual?	1 | 2 | 3 | 4 | 5	
Q8	Is school fun?	1 | 2 | 3 | 4 | 5	
Q9	Are you excited to go to school?	1 | 2 | 3 | 4 | 5	
Q10	Do you enjoy school?	1 | 2 | 3 | 4 | 5	
Q11	Are you happy at school? How much?	1 | 2 | 3 | 4 | 5	
Q12	Are you good at using robots?	1 | 2 | 3 | 4 | 5	
Q13	Is learning about robots fun?	1 | 2 | 3 | 4 | 5	
Q14	Do you want to learn more about robots?	1 | 2 | 3 | 4 | 5	
Q15	Are you interested in science, technology, engineering, and/or math?	1 | 2 | 3 | 4 | 5	
Q16	Are you interested in arts (e.g., acting, dancing, music, drawing)?	1 | 2 | 3 | 4 | 5	
Q17	What do you want to do with robots during this program (list three things)?		
Q18	If you have robots, what are the things that you want to do with the robots?		
Q19	Have you ever seen robots (in person or on video)? If so, where/when?		
Q20	What do robots look like (color/shape/size)?		
Q21	What are the things that robots can do?		
Q22	How to make robots act/sing/dance/draw?		
Q23	Here are the pictures of robots. Can you pick the robots that can…?	1. change their facial expressions (happy/sad face)	Milo | Nao | Pepper | Aibo |Quincy
		2. talk	
		3. walk	
		4. dance	
		5. draw	
		6. reply back to you	
		7. order them from the most favorite (1) to the least favorite	

### Entity questions

Through structured interviews, the entity questionnaire compared children’s perceptions of social robots, beetles, and babies in various mental capacity items (Weisman, 2022). The items included Affective Experience, Perceptual Abilities, Physiological Sensations, Cognitive abilities, Agentic Capabilities, Social Abilities, and the Other item (“be aware of things”) (Q2 - Q4 in Table 2). Results were analyzed using a 2 (Time) x 3 (Entity Type) repeated measures Analysis of Variance (ANOVA) with a Bonferroni adjustment. The responses were measured ordinally (No = 0, Kinda = 0.5, and Yes = 1). However, to observe the interaction between the two variables, we used repeated measures ANOVA for the analysis rather than non-parametric methods Previous studies have suggested that F-test, commonly used in ANOVA, was resilient to violations of the interval data assumption and suitable for conducting statistical tests on data from scales without resulting in bias (Carifio and Perla, 2007; Norman, 2010). The missing data for the entity questions were replaced with the mean values of corresponding items (Downey and Craig, 1998). As we were not interested in how children perceive all three entities (robots, babies, and beetles) collectively before and after the program, the results for the time variable are not presented here. The interaction terms reflect the change in their perception of entity types before and after the program.

**FIGURE 5:**
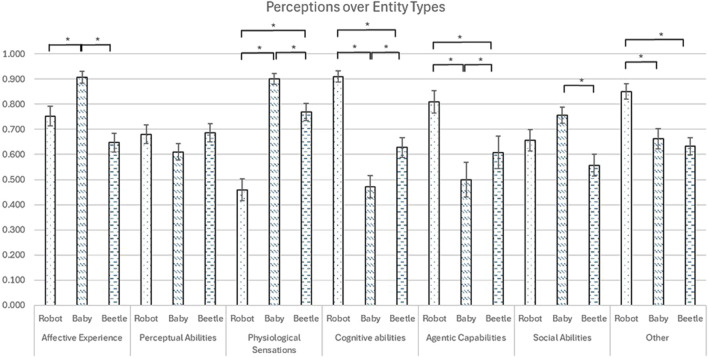
Perceptions of entity types (*: *p* < 0.05 with Bonferroni adjustments).

The results showed the main effects of entity type and interactions between time and entity type (*N* = 21). The main effects of entity type were observed across all items except for Perceptual Abilities (Table 3). Participants rated robots significantly higher than babies and beetles in Cognitive Abilities, Agentic Capabilities, and “Awareness of Things”. Babies received significantly higher ratings than robots and beetles in Affective Experience and Physiological Sensation. Babies were also rated significantly higher than beetles in Social Abilities. However, beetles received significantly higher ratings than babies in Cognitive Abilities and Agentic Capabilities (Figure 5).

**TABLE 3:** Statistics for perceptions of entity type [*Mean(SD)*; *: *p* < 0.05].

	Entity Ty*p*e			
	Robot	Baby	Beetles	Sig.
Affective Experience	0.76(0.18)	0.91(0.11)	0.65(0.18)	*F*(2,40) = 17.89, *p* < 0.01^*^, *η*_*p*_^*2*^ = 0.47
Perceptual Abilities	0.69(0.17)	0.62(0.16)	0.69(0.17)	*F*(2,40) = 1.63, *p* = 0.22
Physiological Sensation	0.46(0.20)	0.91(0.10)	0.77(0.16)	*F*(2,40) = 48.31, *p* < 0.01^*^, *η*_*p*_^*2*^ = 0.71
Cognitive Abilities	0.91(0.10)	0.48(0.21)	0.63(0.18)	*F*(2,40) = 44.10, *p* < 0.01^*^, *η*_*p*_^*2*^ = 0.69
Agentic Capabilities	0.82(0.21)	0.50(0.32)	0.61(0.30)	*F*(2,40) = 10.03, *p* < 0.01^*^, *η*_*p*_^*2*^ = 0.33
Social Abilities	0.66(0.20)	0.76(0.15)	0.56(0.20)	*F*(2,40) = 6.65, *p* < 0.01^*^, *η*_*p*_^*2*^ = 0.35
“Be aware of things”	0.86(0.15)	0.67(0.19)	0.64(0.16)	*F*(2,40) = 14.90, *p* < 0.01^*^, *η*_*p*_^*2*^ = 0.43

**FIGURE 6:**
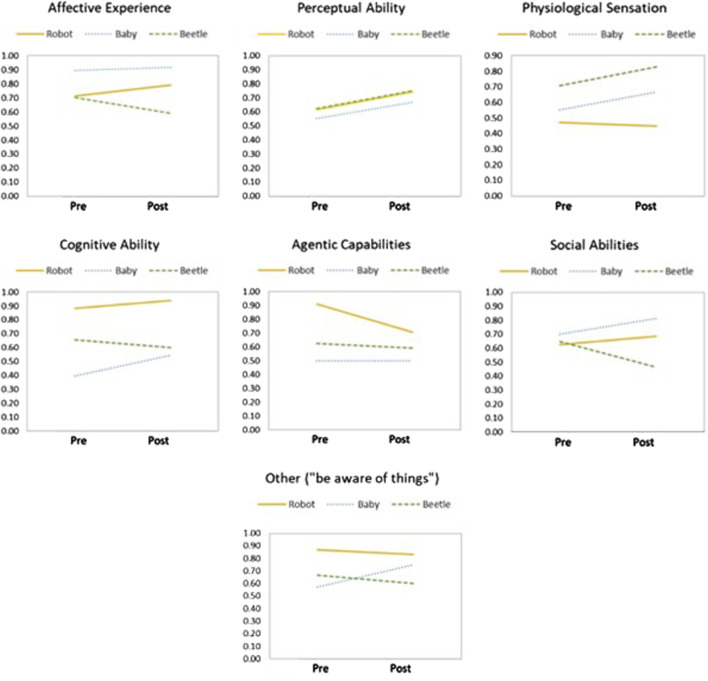
Interaction plots of time and entity types for affective experience, perceptual abilities, physiological sensation, cognitive abilities, agentic capabilities, social abilities, and awareness of things.

Significant time x entity type interaction effects were found in Physiological Sensation, Social Abilities, and “Being Aware of Things” (Table 4). Specifically, children’s ratings for both robots and babies in Affective Experience, Perceptual Abilities, Cognitive Abilities, and Social Abilities increased from before to after the program; however, the opposite trends were observed in Physiological Sensation, Agentic Capabilities, and “Awareness of Things” (Figure 6). A simple effects analysis was performed to compare how children’s ratings of each entity changed before and after the afterschool program. No interaction effects were found in robots before and after the program. Given that comparing babies and beetles was not the focus of our study, we conducted a planned contrast test focusing on robots, which revealed that robots were rated significantly lower post-program than pre-program (*p* = 0.01).

**TABLE 4:** Statistics for perceptions of entity type by time [*Mean(SD)*; *: *p* < 0.05].

Time X Entity Ty*p*e							
	*P*re - Robot	*P*ost - Robot	*P*re – Baby	*P*ost - Baby	*P*re - Beetles	*P*ost - Beetles	Sig.
Affective Experience	0.71(0.22)	0.79(0.25)	0.89(0.18)	0.91(0.12)	0.70(0.24)	0.59(0.26)	*F*(2,40) = 2.58, *p* = 0.09
Perceptual Abilities	0.61(0.19)	0.74(0.19)	0.55(0.21)	0.66(0.13)	0.62(0.19)	0.74(0.22)	*F*(2,40) = 0.04, *p* = 0.97
Physiological Sensation	0.47(0.22)	0.44(0.26)	0.82(0.19)	0.97(0.05)	0.70(0.21)	0.82(0.15)	*F*(2,40) = 4.15, *p* = 0.03^*^
Cognitive Abilities	0.88(0.17)	0.93(0.11)	0.39(0.26)	0.54(0.26)	0.65(0.24)	0.60(0.25)	*F*(2,40) = 2.80, *p* = 0.08
Agentic Capabilities	0.91(0.17)	0.70(0.33)	0.50(0.41)	0.50(0.35)	0.62(0.40)	0.59(0.34)	*F*(2,40) = 1.71, *p* = 0.20
Social Abilities	0.62(0.25)	0.68(0.24)	0.70(0.20)	0.81(0.18)	0.64(0.32)	0.46(0.23)	*F*(2,40) = 4.97, *p* = 0.02^*^
“Be aware of things”	0.86(0.17)	0.83(0.18)	0.57(0.32)	0.75(0.17)	0.66(0.23)	0.60(0.22)	*F*(2,40) = 4.09, *p* = 0.03^*^

### Children’s opinions about robots

We interviewed students (*N* = 9) with five open-ended questions about their opinions about robots before and after the program. The open-ended interview questions were as follows:

• What do robots look like (color/shape/size)?
• What are the things that robots can do?
• How do robots act/sing/dance/draw?
• What do you want to do with robots during this program (list three things)?
• If you have robots, what are the things that you want to do with them?

**FIGURE 7:**
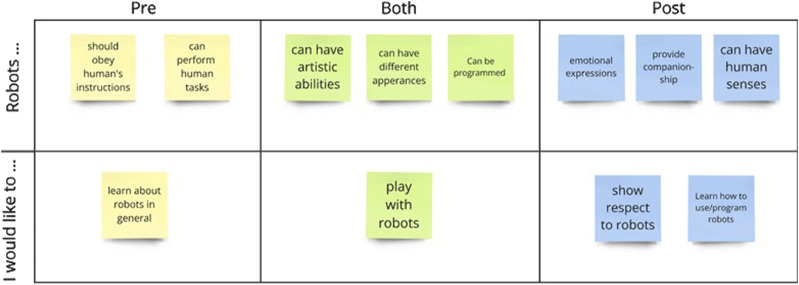
The affinity diagram of children’s opinions of robots.

We analyzed children’s responses through the affinity diagram (Holtzblatt and Beyer, 2017), in which a researcher grouped students’ responses based on observed patterns. The affinity diagram is a simple yet flexible qualitative data analysis method that does not require coding but instead categorizes responses into similar concepts (Holtzblatt and Beyer, 2017). We used this approach for children’s interview data for the open-ended questions because their responses were mostly short phrases, which was not sufficient for a conventional thematic analysis method. We developed two main ideas from children’s responses, shown in the rows in Figure 7: 1) their perceptions of robots’ capabilities (“Robots … ”) and 2) their attitudes toward interacting with robots (“I would like to … ”). Then, we compared the changes in children’s responses to these two topics before and after the program; in addition, we reported the common concepts from both time points. To conduct this bottom-up analysis, we transcribed children’s responses and generated concepts by reviewing their utterances individually. Each block in Figure 7 represents a concept generated from children’s responses. The direct quotes from children are discussed in detail below.

Children’s responses revealed a change in their attitudes toward robots before and after participating in the afterschool program. Before the program, children considered themselves to have higher authority than robots. For example, students mentioned that robots should “do whatever I say,” “do what I tell them to do,” work “under my command,” and “make them clean my room.” After the program, fewer children discussed robots as tools and showed a sense of caring and respect when discussing robots in the interview, such as robots should “do what they (robots) want to or can do” and “don’t want to be mean” to robots. Another notable difference in children’s responses was their thoughts about the robots’ capabilities and responsibilities. In the pre-program interview, children discussed that robots could perform a variety of complex tasks, such as “grabbing”, “picking up stuff,” “flying,”, “cooking,” “driving,” and so on. In contrast, in the post-program interview, children’s responses became more factual and focused more on the affective aspect of robots. Children mentioned that robots could feel different emotions, such as “be happy,” “be proud,” “be embarrassed,” and “feel loved.” Children also thought that robots could provide them with companionship, such as “hugging them” and “playing video games with them.” Moreover, one child stated that robots provide them with protection because robots could “act like a bodyguard.” Finally, children showed a higher curiosity in “learning about robots” after the program because their responses included specific topics such as “making robots,” “coding/programming robots,” learning about “how robots work,” and “robot projects.”

Children’s responses remained consistent before and after the program when discussing the artistic abilities and appearance of robots. They mentioned that robots can “dance,” “sing,” “play music,” and “draw,” and robots can be in “any color and any shape.” However, a few children mentioned that robots are “grey” and made of “metals,” corresponding to the appearance of NAO and Pepper. Lastly, children maintained their interest in interacting with robots before and after the program, as they would like to “play with robots” and “be friends.”

### Children’s interest, self-efficacy, and curiosity in robots and STEAM

**FIGURE 8:**
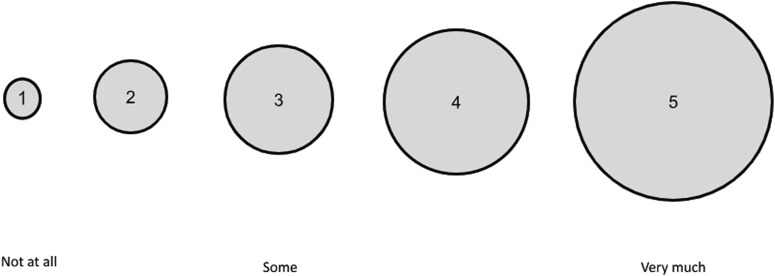
Five circles to help children understand the likert scale.

Children’s interest, self-efficacy, and curiosity about robots and STEAM were measured before and after the program using a 5-point Likert scale with structured interview questions (Table 2). To help children become familiar with the Likert scale, we presented five circles of different sizes for comparison (Figure 8). Children’s interest in STEAM was measured with two items (i.e., “Are you interested in science, technology, engineering, and/or math?” and “Are you interested in arts?”). Children’s interest was measured by the question, “Is learning about robots fun?”, and self-efficacy was measured by the question, “Are you good at using robots?” (Master et al., 2017). Children’s curiosity measure was measured by the questions, “Do you want to learn about robots?” for the pretest or “Do you want to learn more about robots?” for the posttest, adapted from the Children’s Science Curiosity Scale (Harty and Beall, 1984). Children’s pre- and post-program responses to these questions were analyzed using paired samples t-tests. No significant differences were found (Table 5).

**TABLE 5:** Statistics for children’s interest, self-efficacy, and curiosity in robots and STEAM [*Mean(SD)*].

	Time		
	*P*re	*P*ost	Sig.
Interest in STEM	3.86(0.42)	4.14(0.59)	*t*(1,20) = 0.39, *p* = 0.70
Interest in Arts	4.21(0.33)	4.71(0.47)	*t*(1,20) = 0.86, *p* = 0.40
Self-efficacy	4.57(0.28)	4.71(0.40)	*t*(1,20) = 0.29, *p* = 0.77
Curiosity	3.85(0.33)	4.86(0.46)	*t*(1,20) = 1.76, *p* = 0.09

### Teacher interview

After the program, four teachers were interviewed about their attitudes toward using emerging technologies, such as robots, in informal educational settings and their thoughts about the present afterschool program (Table 6). The interview transcripts were analyzed through a qualitative method called thematic analysis, in which the responses were coded and categorized into different themes (Braun and Clarke, 2012). Three researchers were trained to conduct the thematic analysis of the teacher interview results to ensure a systematic approach. First, each interviewee’s sentences were separated into utterances by punctuation. Then, two of the researchers individually coded the utterances and developed themes based on the patterns observed from the transcript and codes. Finally, the third researcher compared each researcher’s themes and decided on the final theme for each utterance to resolve conflicts. The following four themes emerged from the teacher interview data:

**TABLE 6:** Teacher interview questions.

#	Question
1	A. What excited students most about the child-robot theater afterschool program? B. Can you elaborate on the word “interacting”? C. What are some of the least exciting things for students about the child-robot theater afterschool program?
2	How engaged were the students in the after-school program?
3	How motivated were the students to take part in STEAM-related activities in the program?
4	How have you heard students describe the experience to other students, teachers, and parents about this program?
5	What was helpful for the children to learn?
6	Have you seen evidence that they learned anything from the program? (like STEM, art, etc.)
7	A. Which module do you think the children learned the most? B. Which module do you think the children learned the least?
8	A. Which module do you think children were engaged in the most (enjoyed the most)? B. Which module do you think children were engaged in the least?
9	After completing this program, what are your thoughts on using robots in STEM education or art education?
10	Are you more or less likely to use robots in future afterschool programs?
11	How might robots be used while teaching or learning? What activities would you like to do in your class using robots?
12	What do you think about having robots in the classroom during the school day? Do you have any expectations, suggestions, or concerns?
13	What do you think about using robots in future afterschool programs?
14	Do you have any suggestions for the future of the Robot Theater Program? What can we do better next time?
15	What's your experience teaching or working with children?
16	How long actually have you been here as a teacher?

### Theme 1: Children’s positive attitudes toward human-robot interaction

Throughout the program, teachers noticed that children were naturally curious and excited about interacting with robots in the afterschool program. Teachers reported that children expressed a strong desire to interact with robots during the afterschool program such that “all the kids wanted to sign up” and “they are so excited to see y’all at the door”. Children would “ask the teachers all kinds of questions about the program,” “raise hands to answer questions” during the program, and “they are learning while having fun.” In addition, children were reluctant and even upset to leave the class when the session ended or when their parents picked them up early. Teachers mentioned that a few children asked their parents to pick them up later to have more time to interact with the robots, otherwise, they would “throw a tantrum because they want to stay longer” and “they never want to leave.”

Children’s enthusiasm for the afterschool program was also evident outside of the sessions. Teachers observed that children shared their experiences with their parents, peers, and staff. A teacher noted that one of the parents said, “The program is all the child wants to talk about.” Additionally, teachers mentioned that children shared their learning experiences with them during the day, such as “remembering the story and robots’ names.” Teachers stated that this curiosity would help students develop their creativity and problem-solving skills, while also increasing their familiarity with robots.

### Theme 2: robots and modularized lessons foster engaging learning experiences for children

Teacher interview data suggest that robots can provide joyful learning experiences for children through engaging and entertaining interactions, encouraging a positive approach to education and technology. Teachers noticed that children enjoyed the afterschool activities, especially when they mentioned specific modules that help students learn arts-related elements. For example, teachers mentioned that children enjoyed the drawing and dancing modules because it was an “eye-opening” experience for the students. Displaying the robots was one of the attractive points of the program, and students “never complain” about the learning experience because “they talked about it with a lot of excitement.” Having robots display input from children as they wanted was among the students’ favorite aspects of the program. Overall, teachers used keywords such as “enjoyed,” “excited,” and “loved” during the interviews to describe children’s experiences, suggesting children’s enthusiasm and satisfaction with the afterschool program.

### Theme 3: Teachers’ positive experience with teaching using emerging technology

The present afterschool program reminded the teachers of their own positive experiences with technology at a young age as they observed children interacting with robots. One of the teachers mentioned that they were “shocked” by how students could pick up so quickly on playing with the robots because they did not have one growing up. In addition, they noted that not only did children enjoy the program, but all of the teachers and staff learned about robots from the program. Most of the teachers expressed their fondness for the robots as they “would love this when they were kids.” Even one teacher stated, “If we could afford to buy robots to help start my own program, I would love that,” and they believed “robots are essential in everyday life.” This optimistic view from teachers can encourage them to incorporate technology into their teaching methods and recognize its potential in future educational environments.

### Theme 4: Balancing the use of robots in educational settings

Emerging technologies, such as AI tools and robots, to facilitate learning activities benefited young learners in the afterschool program. Teachers stated that robots increased students’ curiosity and helped them absorb STEAM-related knowledge through embodied learning with the physical presence of the research robots. For example, teachers mentioned that the robots “demonstrated how to do different things, such as programming,” that students could “interact with the screen (Pepper’s tablet)”, and that “I can definitely see them learning and gaining all kinds of different knowledge and experiences from the program.” In this afterschool program, students could immediately see the results of their programming on the robots in real-time. The program also taught students how to interact with robots appropriately, considering their capabilities and limitations during hands-on activities, as teachers mentioned that “it is really cool that children have the concerns of how they need to treat the robots as if they are actually taking care of it” and “students knew how to move Pepper’s arms and showed different skills.” With robots’ assistance, teachers mentioned that students, especially the autistic children who participated in the program, were able to learn emotional and social skills through human-robot interactions because “the eye contact of the robot dog (Aibo) and things like that really help children” with “practice social, emotional skills”. Even two teachers mentioned, “We have an autistic child, and he was playing with the dog, and he really was following the cues like eye contact.”

Despite the benefits of using robots in the learning environment, teachers mentioned that there could be several downfalls. The distraction created by robots was a major drawback of this type of embodied learning. Children often were too excited about robots. Hence, they focused more on being curious about this new technology itself rather than on learning the fundamentals of STEAM. Teachers were concerned that robots could be “a big distraction” because students were “too focused on robots they might not really learn what you guys are presenting.” Because of children’s enthusiasm for using robots, teachers observed a strong desire among them to interact with robots, which might negatively influence their relationships with peers. A couple of teachers mentioned, “I know kids get really carried away with robots. They all want to fight like and be really aggressive.” These responses suggest that incorporating robots into educational settings requires complex logistics. Teachers mentioned that training is required for teachers and staff and that preparing robot setups could be time-consuming, such as charging the robots to prevent power loss during educational activities. Teachers and staff noted that they must pay attention to students during interactions given the high cost of the robots. This discussion also raised the later concern of accessibility, whether all schools “have the money for it (using robots in educational settings).” Robots can serve as valuable educational tools to provide children with an engaging learning experience; however, if not carefully integrated into educational environments, robots can create distractions and competition for resources that divert children’s attention from learning objectives.

## Discussion

The present study developed and implemented a 9-week child-robot theater afterschool program to promote STEAM education in elementary school children using social robots. The study examined children’s perceptions of robots compared to other entities (i.e., babies and beetles) before and after the program. Children also completed interviews with structured and open-ended questions about their learning interests and options about robots before and after the program. In addition, teachers were interviewed regarding their observations of students and perceptions of using robots in educational settings after the program. The results showed that 1) children perceived robots to have significantly higher intelligence than babies, but lower emotional intelligence than babies; 2) children perceived robots to have significantly less Physiological Sensation, Agentic Capabilities, and Awareness of Things after attending the program; 3) children’s attitude towards robots shifted from viewing them as entities that follow commands to recognizing them as beings that need respect and care, as evidenced by changes in their responses to the open-ended interview questions before and after the program; 4) children’s creativity in describing robots’ appearance did not change throughout the program, and they perceived robots to have artistic skills, such as drawing and dancing both before and after the program; and 5) teachers observed children’s enthusiasm in interacting with robots and believed that using robots enhanced children’s learning experiences, but they also raised concerns about the negative impact of using technologies in educational settings.

### Children’s responses to pre- and post-program entity questions

Based on the students’ responses to the entity questions, students might perceive robots as more intelligent than babies because their ratings for robots were significantly higher than those in Cognitive abilities, Agentic Capabilities, and Awareness of Things in general. In the study by Williams et al. (2019), young children also perceived robots as smarter than themselves. The robots used in the present study might have perceived as competent, as they were programmed with speech and movements that showed awareness of the environment and exhibited cognitive skills (Baumann et al., 2023). However, the students rated babies significantly higher scores in Affective Experience and Physiological Sensation than robots. These findings align with prior research suggesting that children might perceive robots as less emotionally and physiologically complex than humans despite any human features (Manzi et al., 2020). Despite being programmed with emotional expressions throughout the afterschool program, children still perceived human babies as more capable of providing affective responses and using physiological senses than robots.

Regarding the interaction effects between time and entity type, the results showed that children’s perception of robots and babies increased together in Affective Experience, Perceptual Abilities, Cognitive Abilities, and Social Abilities after participating in the afterschool program. Although children perceived robots to have significantly lower affective capabilities than babies, the qualitative part of the study revealed that children still believed that robots could have emotions (affective experience), human senses (perceptual abilities), the ability to think (cognitive abilities), and the ability to recognize others’ emotions and empathize (social abilities) after participating in the program. These findings may imply that children learned about the human and social aspects of robots and applied similar perceptions to the robots. Applying social concepts to robots may encourage children in learning activities. This perceptual adjustment may help children become familiar with the idea that robots are entities capable of learning, interacting, and even displaying limited emotional awareness through cues, such as facial expressions and interactive conversations (Chen et al., 2020; Rohlfing et al., 2022; Broekens, 2007). Previous studies have also shown that when robots were programmed with behaviors and emotions that mimic human interactions and behaviors, children showed more acceptance in building relationships with the robots (Kanda et al., 2012). These findings align with teachers’ observations, in which children showed a willingness to learn and interact with robots throughout the program.

However, children’s ratings of robots’ Physiological Sensation, Agentic Capabilities, and Awareness of Things decreased after the program, whereas the ratings of babies increased or remained the same. Young children may have developed the ability to differentiate robots from humans and do not attribute biological functions to robots, even when robots displayed human-like characteristics during the afterschool program. Although children perceived robots to have significantly higher scores in Agentic Capabilities and “be aware of things” than babies in general, children’s ratings for robots in those items decreased after the afterschool program. Specifically, children rated robots significantly lower in Agentic Capabilities after the program than before the program. Prior research has found that young children usually give generous ratings for robots’ decision-making abilities initially, assuming robots’ programmed responses were intentional decisions (Williams et al., 2019). After completing the afterschool program, children may have gained more knowledge about the capabilities and limitations of robots, helping them develop a more realistic understanding of robots. This result is consistent with the findings from the study by Reinecke et al. (2025), in which they observed a decline in children’s perception of robots in terms of mental capacities after exposure to the robots. These findings suggest that fostering a balanced perspective of robots is important for children to develop appropriate trust in robots.

### Children’s attitudes and opinions about robots analyzed through affinity diagrams

In the interview responses to the open-ended questions, children believed that robots could have artistic abilities, which can help students expand their understanding of creativity and technology. Integrating arts in robots invites children to use innovative approaches to problem-solving through visual thinking and encourages cross-disciplinary learning experiences (Sullivan et al., 2017; Flatebø et al., 2024). Another notable observation in children’s interview results was that their creativity in answering the question of what they thought a robot should look like remained from the pre-to post-program interviews. We can cautiously state that the present program did not constrain children’s imaginative expression and innovative thinking, even after participating in the program with specific social robots and STEAM topics.

Interestingly, children’s attitudes changed from viewing robots as entities that should listen and follow human instructions in the pre-program interview to entities that need respect and care in the post-program interview. This result may imply that the present program successfully taught children the importance of appropriate human-robot interactions. Many research studies aim to prepare young learners for a world of human-robot coexistence, and appropriate interactions between children and robots are increasingly important to improve children’s ethical awareness and social skills development as well as maintain the sustainability of technologies used in educational programs. Introducing children to respectful interactions with robots at an early age not only prevents children from potential misuse of robot assistance in the future (Tolksdorf et al., 2021) but also improves their ability to learn in school (Silvis et al., 2022; Tanaka and Ghosh, 2011).

The present afterschool program also successfully maintained children’s interest in learning about robots and their willingness to interact with technologies. Although there was no significant difference in children’s interest, self-efficacy, and curiosity about robots and STEAM, children’s ratings remained in the range between 4 and 5 out of 5, indicating that children most likely/strongly agreed that they were interested in STEAM, were good at using robots, and desired to learn more about robots. Children’s ratings slightly increased from the pre-to the post-program interviews; thus, we can cautiously imply that children maintained a high level of learning interests and curiosity throughout the program. This portion of the structured interview results were consistent with the children’s qualitative answers in this study as well as the results of the previous robot theater afterschool program (Dong et al., 2023).

Children’s responses to things they wished to do with robots revealed their enthusiasm for learning about the purpose and operation of robots and the fundamentals of programming to control robots. They also developed an affective bond with robots because of their strong desire for robot companionship during the program. Numerous research studies have shown that having robot companionship and hands-on activities increased children’s motivation and engagement in learning and improved their perceptions of robots (Chen et al., 2020; Park et al., 2019; Wei et al., 2011). From children’s qualitative responses during interviews, the present afterschool program effectively engaged children in learning with technologies without constraining their creativity and educated them about the importance of respectful human-robot interactions.

### Teachers’ interview: opinions about robots and the afterschool program

Teachers’ insights in the interview provided valuable feedback for the present afterschool program. Teachers stated that children showed excitement and enjoyment throughout the program, as evidenced by their shared stories with peers, teachers and staff, and children’s parents. This positive feedback supported the findings that the present afterschool program successfully engaged children in the learning process. In addition, the program’s module objectives resonated well with students and increased children’s willingness to share the experience with others. Not only did the students have an enjoyable experience, but the teachers also expressed their positive envision of using robots in informal learning programs in the future. Previous studies have shown the positive impact of adopting introducing robots into the classroom as teaching assistants on students’ learning interests and outcomes (Han et al., 2010; You et al., 2006). With the exposure to social robots in the present study, teachers were more familiar with technology and expressed their confidence in using robots in their own programs and classes.

Teachers expressed surprise at how quickly students learned the fundamentals of robots and related STEAM topics in the program and recognized the importance of using robots to facilitate embodied learning in educational settings. Throughout the program, robots were programmed to display learning materials to students and to invite children to perform together in the final theater play that incorporated their design ideas using technologies. Children demonstrated their understanding by discussing the robots’ physical capabilities and recalling learning materials, such as the details of the hands-on activities and the programming logistics. More specifically, teachers addressed the benefits of using robots in educational settings to promote the social and emotional skills of students with autism. Research studies have found that robot interventions positively impact social skills training for autistic children (Ntaountaki et al., 2019; Yun et al., 2017). Moreover, a survey by Göngör and Tutsoy (2024) found that a social robot environment could provide effective interactions for children with autism and improve their emotion recognition skills. These findings suggest that embodied learning with social robots may further enhance students’ positive perceptions of their learning experience (Chin et al., 2014), sense of self-development (Ioannou and Ioannou, 2020), and computational thinking skills (Shapiro and Stolz, 2019).

Despite teachers’ positive attitudes toward using robots in educational settings, they also stated their concerns and provided practical suggestions for the afterschool program. Teachers’ concerns were mainly related to the distractions caused by the presence of social robots, the disruptions in peer relationships, and the complex logistics of setting up technologies. Numerous research papers have investigated educators’ concerns about the presence of robots, causing students to focus more on the robots rather than the learning materials (Alhashmi et al., 2021; Perella-Holfeld et al., 2024; Reich-Stiebert and Eyssel, 2016; Sharkey, 2016). However, teachers' concerns have not been fully examined in research studies with embodied learning, while students’ learning outcomes and motivation still showed positive results with the physical presence of robots in previous studies (Alhashmi et al., 2021; Han et al., 2010; You et al., 2006). The presence of social robots may inadvertently lead to undesirable behaviors in specific contexts, such as classroom settings, rather than affecting children’s learning outcomes in general. Maggi et al. (2021) state that a robot with an authoritative interaction style may be more appropriate when the learning task involves high cognitive demands. Future studies should consider the teaching or interaction style of social robots while designing and programming them for educational programs with the consideration of specific contexts and learning objectives.

Teachers and researchers noticed students’ negative interpersonal behaviors with their peers while interacting with robots during the free-play session. This was reflected in students’ aggressive tone when communicating with peers, intense physical contact, and dissatisfaction with the program because of the limited number of social robots in the present program. Teachers mentioned that students fought for the opportunity to have one-on-one interaction with the social robots in the present afterschool program. This finding was consistent with parents’ and educators’ concerns about the negative impact of using emerging technologies in education on interpersonal relationships (Perella-Holfeld et al., 2024). While this observation may indicate children’s strong willingness to interact with robots, it is crucial to avoid the negative impact this behavior may bring on their relationships with peers. Finally, teachers expressed their concerns about the low accessibility of using research robots in the educational program as research robots are high-cost and they need additional knowledge and time to set up such technologies. It is important to note that only four teachers completed the interview during the program, and this small sample size may limit the generalizability of the teacher interview results.

#### Design guidelines

The present study used robots in an educational setting as an embodied learning experience for the children, which fostered an engaging learning experience. The results of the study showed that students perceived robots as significantly more intelligent but with less emotional capabilities than babies. After participating in the afterschool program, students perceived robots as having significantly less Physiological Sensation, Agentic Capabilities, and Awareness Capabilities than babies (RQ1). Throughout the program, students demonstrated a strong willingness to interact with robots, according to their qualitative responses in their interviews. In addition, even though students thought robots have limited emotional capabilities, students believed robots need respect and care after participating in the program (RQ2). From the children’s and teachers’ qualitative interview responses, we can state that the present afterschool program successfully maintained children’s high levels of interest, self-efficacy, and curiosity about robots and STEAM throughout the program (RQ3). Finally, teachers observed children’s engaged learning behaviors with robots and recognized the importance of using robots in education; however, teachers addressed the concerns about distractions and negative impacts on children’s relationship with peers and the difficulties of using technologies in future educational programs (RQ4). Based on the successful outcome of the present study, we developed a list of design recommendations for future educational programs that use robots in informal learning environments:

• Form a multidisciplinary team: During the development stage of the present afterschool program, our team involved multiple stakeholders from different backgrounds, including industrial and systems engineers, computer scientists, human development experts, and an education administrator. The roles and responsibilities of the industrial and systems engineers and computer scientists were to program robots to facilitate lesson plans and instruct children on STEAM-related topics during the program. The human development experts conducted a literature review and developed age-appropriate questionnaires with relevant questions according to the study goals. The education administrator facilitated effective communication between parents and the team to ensure program implementation. With a cross-functional team, we ensured a comprehensive approach to the study design by involving everyone in the lesson design, holding weekly follow-up meetings to reflect and discuss each week’s progress, and improving the afterschool program through iterations. Having multiple team members was beneficial for data collection, as the present study included interview questions to collect both quantitative and qualitative data, which required a considerable amount of time. A previous child-robot theater afterschool program also addressed the importance of having a multidisciplinary team (Dong et al., 2023).
• Develop modularized lessons: Modularizing the curriculum into four themes provided different learning objectives, allowing the research team and students to focus on specific STEAM topics in depth. With modules tailored to different topics every 2 weeks, students were engaged in the learning experiences with new challenges (Dong et al., 2023). In addition, students could retain their knowledge throughout the program by using the module themes as memory anchors.
• Plan for program logistics: Executing an effective child-robot afterschool program requires thorough logistical planning, such as keeping track of equipment, securing learning space, and saving time for the robot setup (including charging and powering up robots). Providing robot training to each research team member is important as they need to have skills in using the robots to address students’ questions, maintain students’ learning engagement, and monitor inappropriate behaviors.
• Involve teachers and staff in the experiences: Teachers and staff stated their desire to experience this afterschool program, especially if they were students themselves. In addition to assisting with roll call and classroom discipline, teachers and staff could have also more actively participated in the program to learn about robots and encouraged to help answer students’ questions. We found it to be important to involve teachers and staff in the learning process as it familiarizes them with robots. As robots become increasingly more accessible, trained teachers and staff can promote and support the continuity of using emerging technologies, such as robots, within educational environments.
• Minimize distractions from robots: Throughout the program, the research team and the teachers observed that a few students paid more attention to robots rather than to the learning aspects of the program. Future programs should consider strategies to minimize distraction and ineffective learning experiences with robots. One possible solution is to introduce robots later in the free play period, rather than having them in front of children from the beginning of the session.
• Incorporate other low-fidelity technologies: With the limited number of research robots, the present afterschool program also provided low-fidelity robot toys and laptops with AI games for children to continue their interaction with technologies while waiting for their turn to interact with social robots. This encouraged students to stay motivated to learn while applying knowledge using similar tools. We also used a smart projector provided by the school and speakers to present module lessons through visual and auditory interactions.

#### Limitations and future work

There were a few limitations to the present afterschool program that need to be considered in future studies. First, children’s fluctuating attendance posed a challenge to consistent data collection in the present study. Students were not required to participate in the study for the entire program and could attend and leave at any time. This inconsistent nature of the attendance may have influenced the students’ responses to interview questions, as children may not have participated in every session of the program. To improve the data collection process, future studies can conduct a controlled experiment that ensures children’s attendance and a more standardized data collection process. Second, the low accessibility of research robots in educational settings may pose a challenge for other educators and researchers when attempting to replicate the current program in the future. Children were not able to interact with the research robots all the time as they needed to take turns during the program. The present studies compensated for this challenge by providing children with other low-fidelity robot toys that enabled continuous interactions with robots. Future research studies should consider having additional resources or equipment that allow children to access emerging technologies during their learning experiences.

Third, the present program developed a storybook to help students learn about robots’ physical capacities and expressions. However, the framing of the storybook might have created a potential bias in children. Such framing might have lead children to believe that robots have significant emotional and social capabilities, such as having emotional expressions and making friends with each other, influencing ed children’s perception and conceptualization of robots. The present study mitigated this influence by discussing different robot characteristics and limitations rather than focusing solely on the emotional and social aspects. Future programs should avoid the potential impact of environmental variables that might influence students’ perceptions. Fourth, the wide age range of children who participated in the current study could have led to significant variability in children’s responses due to differences in children’s cognitive developmental stages (Huitt and Hummel, 2003). For instance, according to Piaget’s theory, children in the pre-operation stage (2–7 years old) exhibit intuitive thoughts, whereas older children in the concrete operation stage (7–11 years old) are likely to engage in more organized and logical thinking (Wadsworth, 1996; Huitt and Hummel, 2003). Given the age difference, younger children may struggle more than older children with interview questions that require complex reasoning and self-reflection. When children needed more clarification during interviews, the researchers rephrased the questions using language tailored for children and provided examples to ensure that all students provided responses based on their own perceptions and reasoning. Future studies should consider balancing groups based on children’s age ranges to collect more consistent data.

Another challenge in conducting the 1:1 interview with children was that the interviewer’s characteristics and style could influence the children’s responses. In the present study, we ensured consistency in interview procedures by establishing protocols and discussing how to interact with children using the Virginia Tech Center for Educational Networks and Impacts (CENI) recommendations. In addition, children’s interviews were conducted during the same time, during free play sessions, to ensure consistency. Although we sought a quiet location for the interviews, background noise and the presence of other children, even if not in proximity, may have caused distractions. Future studies should minimize the variability introduced by individual researchers and control other environmental factors, such as having consistent data collection procedures and securing quiet and distraction-free environments for interviews. Finally, teachers raised ethical concerns about using robots in educational settings, as a few of the children fought to interact with research robots in the program, which could lead to negative interpersonal interactions with their peers. Future studies should address ethical considerations and investigate how the use of robots influences children’s moral development.

## Conclusion

The present afterschool program was developed with the research objectives of 1) promoting STEAM education in young learners by providing an innovative and engaging learning opportunity using social robots and 2) understanding the impact of embodied learning using social robots in shaping children’s conceptualizations and perceptions of robots, children’s learning experiences, and teachers’ perceptions of using emerging technologies in educational settings. The present study used a mixed-methods approach that examined children’s responses from both quantitative and qualitative analyses providing detailed interpretations of children’s perceptions of robots and experiences in the program. Children’s willingness and excitement to interact with the research robots throughout the program were an indicator of the successful development and implementation of the present child-robot afterschool program. The findings of the study expanded the knowledge of child-robot interactions and provided design recommendations for researchers and educators in developing future educational programs using robots for embodied learning.

## Data Availability

The raw data supporting the conclusions of this article will be made available by the authors, without undue reservation.
